# *Stenosoma stephenseni* sp. n. (Isopoda, Idoteidae), from the southwestern Mediterranean, with a note on the nomenclatural status of Synisoma Collinge, 1917

**DOI:** 10.3897/zookeys.141.1376

**Published:** 2011-10-28

**Authors:** António Múrias dos Santos, Raquel Xavier, Saliha Zenboudji, Tristão Branco, Madalena Branco

**Affiliations:** 1 CIBIO, Centro de Investigação em Biodiversidade e Recursos Genéticos, Campus Agrário de Vairão, 4485-661, Vairão, Portugal; 2 Faculdade de Ciências da Unversidade do Porto, Departamento de Biologia, R. Campo Alegre s/n, 4169-007 Porto, Portugal; 3 Faculté des Sciences, Université de Montepellier 2 Sciences et Techniques du Languedoc, Place Eugène Bataillon, 34095 Montpellier cedex 5, France; 4 Rua de Camões, 788, 2ºD. 4000-142 Porto, Portugal

**Keywords:** new species, North Africa, Mediterranean, Alboran Island, Idoteidae

## Abstract

Recent collections of isopods in Alboran Island and Algeria included several specimens of the species *Stenosoma stephenseni* **sp. n.** This is the fourteenth species described in the genus *Stenosoma* Leach, 1814. Examination of two specimens collected during the Danish oceanographic cruises of the Thor (1908–10) close to the Galite Islands, and identified as *Stenosoma acuminatum* Leach, 1814, revealed that both belong to *Stenosoma stephenseni* **sp. n.** In light of these findings, the Mediterranean records of *Stenosoma acuminatum* are revised, and it is proposed that *Stenosoma acuminatum* is a strictly Atlantic species. An updated diagnosis for the genus *Stenosoma* is given, together with a key for the identification of its species. The nomenclatural status of the name *Synisoma* Collinge, 1917 is addressed, and although it is in prevailing usage, it is shown that *Stenosoma* Leach, 1814 is the valid name of the genus.

## Introduction

In his work on the isopods collected during the Danish oceanographic cruises of the Thor (1908–10) in the Mediterranean and the Black Sea, Stephensen (1915) identified three species belonging to the genus *Stenosoma* Leach, 1814 (=*Synisoma* Collinge, 1917): two specimens of *Stenosoma acuminatum* Leach, 1814, from the Galite Islands (northern Tunisia), two specimens of *Stenosoma capito* (Rathke, 1837), from the Aegean Sea, and one specimen of *Stenosoma appendiculatum* (Risso, 1826), from Cabo da Gata (Spain). Stephensen noted that although the two specimens from the Galite Islands agreed broadly with the figures and descriptions of *Stenosoma acuminatum* provided by Dollfus (1896) and Tattersall (1911), they were “somewhat broader” and the abdomen was “[...] by no means so sharply pointed”. Upon dissection, he also noted that “there is a considerably similarity to *Stenosoma capito* (Rathke, 1837), the appendages being, however, far thicker and heavier”.

In his revision of the British Idoteids, Collinge (1917: 752) noted that “Stephensen’s *Stenosoma acuminatum* (Leach), represented by two examples from different localities – is in one case referable to *Stenosoma capito* (Rathke), the other approaching *Stenosoma lancifer* (Leach), but I am inclined to regard it as a different species”. Later, Monod (1925) corrected Collinge’s assertion, noting that the two specimens from Stephensen came from the same locality (Galite Islands) and were actually a new species (referred to by him as “*Synisoma* sp.?”), whilst Stephensen’s *Stenosoma capito* was a good species collected in a different location (Greece). Since then, the status of Stephensen’s *Stenosoma acuminatum* has been addressed by several authors, most of whom suggest that it is indeed a distinct yet undescribed species of *Stenosoma* (e.g., Amar 1957; Prunus and Pantoustier 1976), and that records of *Stenosoma acuminatum* in the Mediterranean are dubious and should be revised (Junoy and Castelló 2003). Although Stephensen’s material has been available from the Zoological Museum of the University of Copenhagen, Denmark (ZMUC) no further attempt was made to clarify the taxonomic status of the two specimens.

While studying the phylogeography of *Stenosoma nadejda* (Rezig, 1989) we received several specimens from the Alboran Island (provided by JM Guerra García). Analysis of the mitochondrial gene cytochrome c oxidase subunit I revealed that the Alboran specimens belonged to a very divergent lineage from “*Stenosoma nadejda*” and were potentially a new species (Xavier et al. 2011). However, because all individuals from this new lineage were *mancas*, no morphological analysis was possible. Successful sampling of adults from Algeria in 2009 allowed us to start a detailed morphological analysis of this lineage. Morphological similarities between these recently collected individuals and Stephensen’s description of *Stenosoma acuminatum*, led us to request the material from the Thor campaign (1908–10), deposited at the ZMUC.

In this work, we describe a new species of *Stenosoma*, in which we include the specimens of Stephensen (1915) from the Galite Islands, and we discuss the implications of this finding on the distribution of *Stenosoma acuminatum* in the Mediterranean. Additionally, we address the nomenclatural status of the name *Synisoma* Collinge, 1917, which is in prevailing usage. An amended key is given for the species of the genus *Stenosoma* based on the one provided by Castellanos and Junoy (2005).

## Material and methods

Specimens were collected on intertidal algae during low tides, in the winter of 2005 (Alboran Island) and the summer of 2009 (Algeria). All specimens were preserved in 96% ethanol. Description is based on the male holotype unless otherwise stated. Body length measured dorsally from midpoint of cephalon’s anterior margin to posterior of pleotelson. The holotype is deposited in the Zoological Museum, University of Copenhagen, Denmark. All taxonomic work is attributed to A. M. dos Santos and R. Xavier.

### Abbreviations

BMNHThe Natural History Museum, London, UK


MNHNPMuséum National d’Histoire Naturelle, Paris, France


ZMUCZoological Museum, University of Copenhagen, Denmark


CIBIO-UPCentro de Investigação em Biodiversidade e Recursos Genéticos, Universidade do Porto, Portugal


ICZNInternational Code of Zoological Nomenclature (ICZN, 1999).


## Taxonomy

**Order Isopoda Latreille, 1817**

**Familiy Idoteidae Samouelle, 1819**

### Genus Stenosoma

Leach, 1814

http://species-id.net/wiki/Stenosoma

Stenosoma Leach, 1814: 433.– Leach, 1815: 365.– Samouelle, 1819: 107.– Desmarest, 1823: 374.– Desmarest, 1825: 290.– Latreille, 1829: 139.– Moore, 1839: 294.– Lucas, 1840: 259.– Hope, 1851: 26.– Dollfus, 1894: 5.– Dollfus, 1896: 54.– Gerstaecker, 1901: 218.– Norman, 1904: 444.– Norman & Scott, 1906: 47.– Tattersal, 1911: 230.– Stephensen, 1915: 15.
Leptosoma Risso, 1826: 107 (no type species designated, see text).– Rathke, 1837: 384.– Lamarck, 1838: 270.– Hope, 1851: 26.
Synisoma Collinge, 1917: 750 (type species *Stenosoma acuminatum*Leach, 1814, by subsequent designation of Kussakin, 1982). Monod, 1923: 97.– Monod, 1925: 70.– Amar, 1957: 74.– Daguerre de Hureaux, 1968: 87.– Naylor, 1972: 46.– Nunomura, 1974: 6.– Prunus & Pantoustier, 1976: 259.– Kussakin, 1982: 184. Brusca, 1984: 107.– Rezig, 1989: 30.– Ormsby, 1991: 758.– Hedo & Junoy, 1999: 88.– Poore, 2001: 221.– Castellanos & Junoy, 2005: 1461.

#### Type species.

*Stenosoma acuminatum* Leach, 1814, by subsequent designation of Kussakin, 1982 (under Article 67.8).

#### Remarks.

The genus *Stenosoma* was described by Leach (1814) simultaneously in two different parts of the Brewster’s Edinburgh Encyclopaedia: in the main section “Crustaceology” (p. 404), and in the Appendix which was published as an integral part of that section (pp. 429–434). Leach (1815) also re-described *Stenosoma* in his popular work “A tabular view of the external characters of four classes of animals, which Linné arranged under Insecta”, a reference erroneously cited as the original description by many authors (e.g. Collinge 1917; Kussakin 1982; Junoy and Castelló 2003). In neither of those publications was a type species designated.

In page 404 of the section “Crustaceology”, *Stenosoma* was clearly described as a tentative subdivision of the genus *Idotea*. After the general description of *Idotea* (numbered as Genus LXIV), Leach (1814) split it into “Genus *Stenosoma* of Leach. ● body linear, external antennae very long” and “●● Body thickest in the middle. *Idotea*, Leach”. In the first division, Leach placed only one nominal species, *Idotea hectica* Pallas, 1772, and in the second division he placed two nominal species, *Oniscus entomon* Linnaeus, 1758 and *Oniscus oestrum* Linnaeus, 1758. The fact that neither “*Stenosoma* of Leach” nor “*Idotea*, Leach” are numbered (as are all other genera in the section) and do not appear either in the list of genera and families at the beginning of the section (as does “Genus LXIV. *Idotea*”, on page 386) or in the marginal notes or the index, shows that at this stage Leach was not yet sure whether genus rank should be accorded to these divisions.

In the Appendix (p. 433), however, *Stenosoma* is re-described as a genus on its own, this time numbered “XI”, immediately after *Idotea* (which is Genus X). There, Leach reformulated the diagnosis of *Stenosoma* (“external antennae longer than the body, the third longer than the fourth joint; body linear”), and included two nominal species, *Idotea hectica* Pallas, 1772 and *Stenosoma acuminatum* Leach, 1814. So, under Articles 12.1 and 12.2.5, the name *Stenosoma* Leach, 1814 is available from page 404, where it is treated as a division of *Idotea* Fabricius, 1798, and from page 433 of the same publication where it is ranked as a genus. Under Article 24.1, precedence must be accorded to the name proposed at higher rank, i.e., *Stenosoma* as a genus, in page 433 of the Appendix. The important point here is that on page 433 Leach included in his genus the nominal species *Idotea hectica* Pallas, 1772 and *Stenosoma acuminatum* Leach, 1814. Therefore, *Stenosoma acuminatum* Leach, 1814 is eligible as type species of *Stenosoma* Leach, 1814 (Articles 67.2 and 67.2.1).

The genus *Stenosoma* was quickly adopted by some leading French zoologists (e.g., Desmarest 1825; Latreille 1829), but others saw no reason to separate the species included within it from the well established genus *Idotea* Fabricius, 1798 (e.g., Milne-Edwards 1840; Bate and Westwood 1868). Meanwhile, congeneric species were being described from the Mediterranean. Risso (1816) described *Idotea lanciformis* from Nice (France) and later (Risso 1826) described two species from the same region in the new genus *Leptosoma* (*Leptosoma appendiculatum* Risso, 1826, and *Leptosoma lanceolatum* Risso 1826), establishing, in part, the diagnosis for the genus that is still in use: the postabdomen (pleotelson) is unarticulated, resulting from the coalescence of the last four pleomeres, without (almost) any trace of segmentation. Rathke (1837) described *Leptosoma capito* from the Black Sea, and Lucas (1849) described *Idotea carinata* and *Idotea angustata* from Algeria. By the end of the 1880s there were at least 11 different species names (in the genera *Stenosoma*, *Leptosoma* and *Idotea*) for idoteids with unarticulated post-abdomen occurring in the North East Atlantic and the Mediterranean.

In his comprehensive monograph of the Idoteidae, Miers (1881) followed the more conservative approach of Milne-Edwards (1840) and Bate and Westwood (1868) and placed in the genus *Idotea* all species described as *Stenosoma* and *Leptosoma*. Later, Dollfus (1896) opted to separate the genera *Idotea* Fabricius, 1798, and *Stenosoma* Leach, 1814, laying the basis for the current taxonomy of this group. He recognized problems with Leach’s oversimplified diagnosis of *Stenosoma* (see above), noting that the taxonomy behind *Leptosoma* Risso, 1826 made it “a better established genus”. Hence, he retained the name *Stenosoma* Leach, 1814 based on precedence, but explicitly used the diagnosis proposed by Risso (1826) to set *Stenosoma* apart from *Idotea*.

In his revision of the British idoteids, Collinge (1917) took a different approach. Based on the wrong assumption that *Leptosoma* Risso, 1826 was preoccupied, and that the name *Stenosoma* had “been used with so many varied conceptions that, with Miers, I agree that it cannot be employed for any section or division of the family” (Collinge 1917: 727), he proposed the replacement name *Synisoma*, together with an emended diagnosis of the genus. Collinge (1917) included two nominal species in *Synisoma* (*Idotea acuminata lancifer* Miers, 1881 and *Stenosoma acuminatum* Leach, 1814) but did not designate a type species for the genus name.

Kussakin (1982) designated *Stenosoma acuminatum* Leach, 1814 (under Article 67.7, cited as “*Stenosoma acuminatum* Leach, 1815”) as type species of *Synisoma* Collinge, 1917. Since that was one of the originally included nominal species, Kussakin’s is a valid subsequent designation. Moreover, because *Synisoma* Collinge, 1917 is a replacement name for *Stenosoma* Leach, 1814 and, as discussed above, *Stenosoma acuminatum* Leach, 1814 is also one of the nominal species originally included in *Stenosoma* Leach, 1814, under Article 67.8 Kussakin’s is also a valid subsequent designation of *Stenosoma acuminatum* Leach, 1814 as the type species of *Stenosoma* Leach, 1814. As for *Leptosoma* Risso, 1826, as far as we can ascertain, no type species has yet been designated, and there is no indication on the present whereabouts of Risso’s type material.

*Synisoma* Collinge, 1917 is currently in prevailing usage, as it has been used virtually in all works published after 1917. To promote nomenclatural stability, the ICZN allows for a reversal of precedence (Article 23.9) whenever a junior synonym is in prevailing usage provided that the two conditions defined in Articles 23.9.1.1 and 23.9.1.2 are both met. In this case, however, the first condition, that the senior synonym has not been used as a valid name after 1899, is not met. In fact, *Stenosoma* Leach, 1814 was used as a valid name in at least six works posterior to 1899: Nobre (1903), Norman (1904), Norman and Scott (1906), Tattersall (1911), Issel (1912), and Stephensen (1915).

Given the complex taxonomic history of the genus *Stenosoma* and its synonyms*,* their diagnoses have been modified on an ad-hoc basis to accommodate each new species described. For example, both Dollfus’ (1894) diagnosis of *Stenosoma* and Collinge’s (1917) diagnosis of *Synisoma* exclude species with an antennal flagellum reduced to a single clavate article. The most recent revision of the genus made by Rezig (1989) did not account for the two Pacific species, *Stenosoma pacificum* (Nunomura, 1974) and *Stenosoma wetzerae* (Ormsby, 1991). Recently, Hedo and Junoy (1999) and Castellanos and Junoy (2005) concluded that the two most important characters distinguishing *Synisoma* (=*Stenosoma*) from the other Idoteidae are a pleon lacking distinct somites and a maxillipedal palp composed of four articles. According to these authors, all other characters display a high degree of intra-generic variability. We hereby present an updated diagnosis for the genus, which is broadened from that given by Rezig (1989).

#### Diagnosis.

Body elongate, lateral margins parallel or sub-parallel, sometimes widening slightly towards pereonites III–IV. Cephalon with pronounced anterolateral lobes, smooth or with a pronounced dorsal tubercle; eyes lateral, small. Antennulae with first article expanded, flagellum composed of a single article. Antenna articles 3–4 longer, flagellum multiarticulated or composed of a single clavate article. Maxillipedal palp with 4 articles. Pereonites smooth, frequently with a shallow dorsal keel, seldom developing into a dorsal triangular tooth; pereonites I–III often with a pair of lateral tubercles. Coxal plates small, round, rarely medium sized and triangular, invisible in dorsal view, or visible dorsally on perionites II–VII or V–VII. Pereopods ambulatory, slender and sub-equal, terminating in a biungulate dactyl with simple setae. Pleon without articulating pleonites (pleotelson), pleonites I–III frequently indicated by incomplete sutures visible ventrolaterally or dorsally (pleotelsonic formula 0+3); pleotelson long, not less than one third of body length, terminally pointed; dorsal surface smooth or with a shallow keel. Penes attached to posterior ventral margin of pleonite 1, fused basally as a penial plate divided over most of its length. Uropod uniramous, endopodite more or less triangular in shape.

#### Species included.

*Stenosoma acuminatum* Leach, 1814; *Stenosoma appendiculatum* (Risso, 1826); *Stenosoma capito* (Rathke, 1837); *Stenosoma carinatum* (Lucas, 1849); *Stenosoma lancifer* (Miers, 1881); *Stenosoma spinosum* (Amar, 1957); *Stenosoma bellonae* (Daguerre de Hureaux, 1968); *Stenosoma pacificum* (Nunomura, 1974); *Stenosoma nadejda* (Rezig, 1989); *Stenosoma mediterraneum* (Rezig, 1989); *Stenosoma wetzerae* (Ormsby, 1991); *Stenosoma raquelae* (Hedo & Junoy, 1999); *Stenosoma albertoi* (Castellanos & Junoy, 2005); *Stenosoma stephenseni* sp. n.

### Stenosoma stephenseni

Santos and Xavier sp. n.

urn:lsid:zoobank.org:act:EDEC2356-58AE-4DBA-B99D-41042CCB0607

http://species-id.net/wiki/Stenosoma_stephenseni

#### Material examined.

*Holotype:* ♂ (13.0 mm, partially dissected, preserved in ethanol 96%), Dellys, Boumerdès, Algeria, 36°55'27.14"N, 3°53'42.30"E, 6 Aug 2009, intertidal seaweeds (ZMUC-CRU-20458).

*Paratypes:* ♂ (12.5 mm), ♀ (11.0 mm), Galite Islands, Bizerte, Tunisia, (approx. 37°31'27.21"N, 8°56'23.54"E), 5 Feb 1909, ‘on the shore' (Stephensen, 1915) (ZMUC-CRU-20228). 2♂ (10.5, 8.9 mm), Dellys, Boumerdès, Algeria, 36°55'27.14" N,3°53'42.30"E, 6 Aug 2009, intertidal seaweeds, (CIBIO-UP, SstDel5 and SstDel9). 3♂ (10.1, 9.9, 10.5 mm), 3♀ (1 ovig. 11.8 mm, 2 non-ovig. 9.1, 9.8 mm), Tighremt, Bejaïa, Algerie, 36°52'0.60"N, 4°51'25.29"E, 4 Aug 2009, intertidal seaweeds (CIBIO-UP, SstTit4, SstTit2, SstTit18, SstTit1, SstTit15, SstTit17, respectively). 2♂ (13.2, 7.9 mm), Sidi Khaled, Tigzirt, Tizi-Ouzou, Algerie, 36°53'48.52"N, 4°10'52.46"E, 28 Jul 2009, intertidal seaweeds (CIBIO-UP, SstTiz16, SstTiz17). 3 mancas (3.8, 4.1, 4.3 mm), Alboran Island, Spain, 35°56'58.06"N, 3°01'48.57"W, 12 Feb 2005, intertidal seaweeds (CIBIO-UP, SstAlb1-3).

#### Diagnosis.

The species is characterised by a smooth and domed cephalon, with a prominent dorsal boss in lateral view; pereonites smooth, lacking lateral tubercles; pereopods II–VII robust, with merus and carpus 1.2 and 1.1 times as wide as long, respectively; pleotelson margins parallel or subparallel, curving regularly towards distal extremity at one third of its length; pleotelson with three pairs of lateral sutures only visible in ventral view; appendix masculina long, extending beyond apical margin of the endopod by more than one fifth of its length, but not beyond apical spines of endopod.

#### Description.

**Figure 1.**
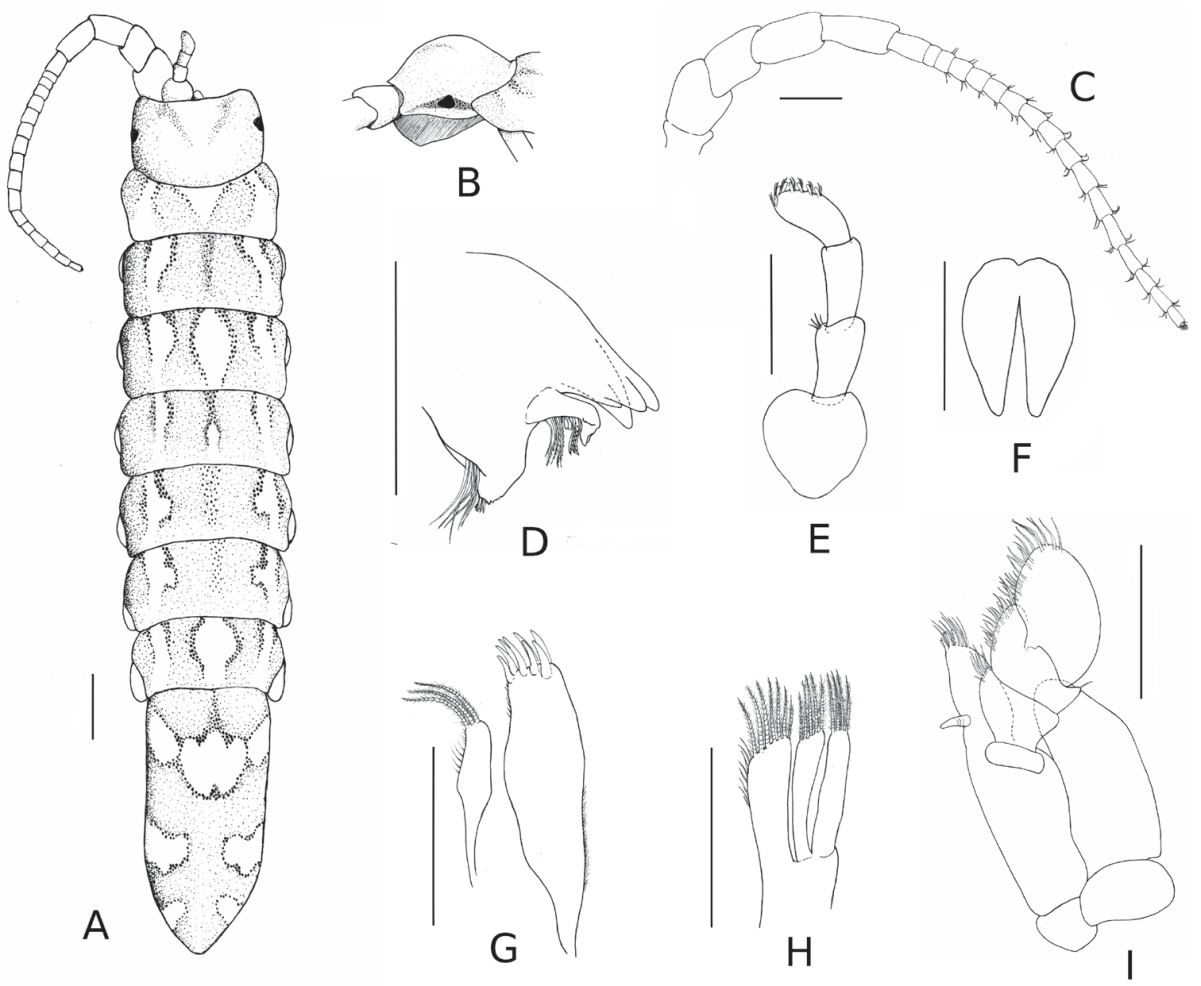
*Stenosoma stephenseni*, sp. n., holotype: **A** dorsal view **B** detail of cephalon **C** antenna **D** left mandible **E** antennula **F** penis **G** maxillule **H** maxilla **I** maxilliped. Scale bars are 500 µm, except for whole specimen (1 mm).

*Body* elongate, five times as long as wide (Figure 1). No secondary sexual dimorphism observable. Length of specimens in type series: 4.3–13.2 mm. Colour light brown to pale yellow, lightly pigmented.

*Cephalon* 1.3 times as wide as long, posterior margin immersed in pereonite I, smooth (no signs of mid-dorsal tubercle) but domed, with a prominent dorsal boss in lateral view; eyes dark, triangular or round, on lateral edge of cephalon; supra antennal line straight, anterolateral angles acute. *Pereonites* smooth, without dorsal carina. Coxal plates small, present on pereonites II–VII and hardly visible in dorsal aspect. All pleonites medially fused, with three pairs of small antero-lateral sutures in ventral view only. *Pleotelson* 2.4 times as long as wide, approximately one third of total body length.

*Antennule*: peduncle of three articles, article 1 ovoid, articles 2–3 cylindrical, similar in size; flagellum bearing seven pairs of aesthetascs. *Antenna*: peduncle of five articles, article 1 reduced, article 2 as wide as long, articles 3–5 progressively longer; flagellum of 17 articles, the distal one with minute vestigial apical article bearing a brush of short setae; flagellum varying from 14 to 17 articles on type series.

*Mandible:* Right mandible incisor 4 toothed; lacinia mobilis with one or two incisors; spine row with seven curved serrate spines; molar process truncate, without tooth. *Maxillule*: inner lobe with three distal plumose spines, inner margin with thin simple setae; outer lobe 1.8 times longer than inner lobe, with eight stout spines, four of them serrate; outer margin with small simple setae. *Maxilla*: trilobate, endopod with seven recurved plumose spines and eight simple setae; inner and outer lobes of exopod with five and four pectinate spines, respectively. *Maxilliped*: palp 4–articulate; exopod round; endite with a single coupling hook, five spines and a few simple setae along the distal margin.

**Figure 2.**
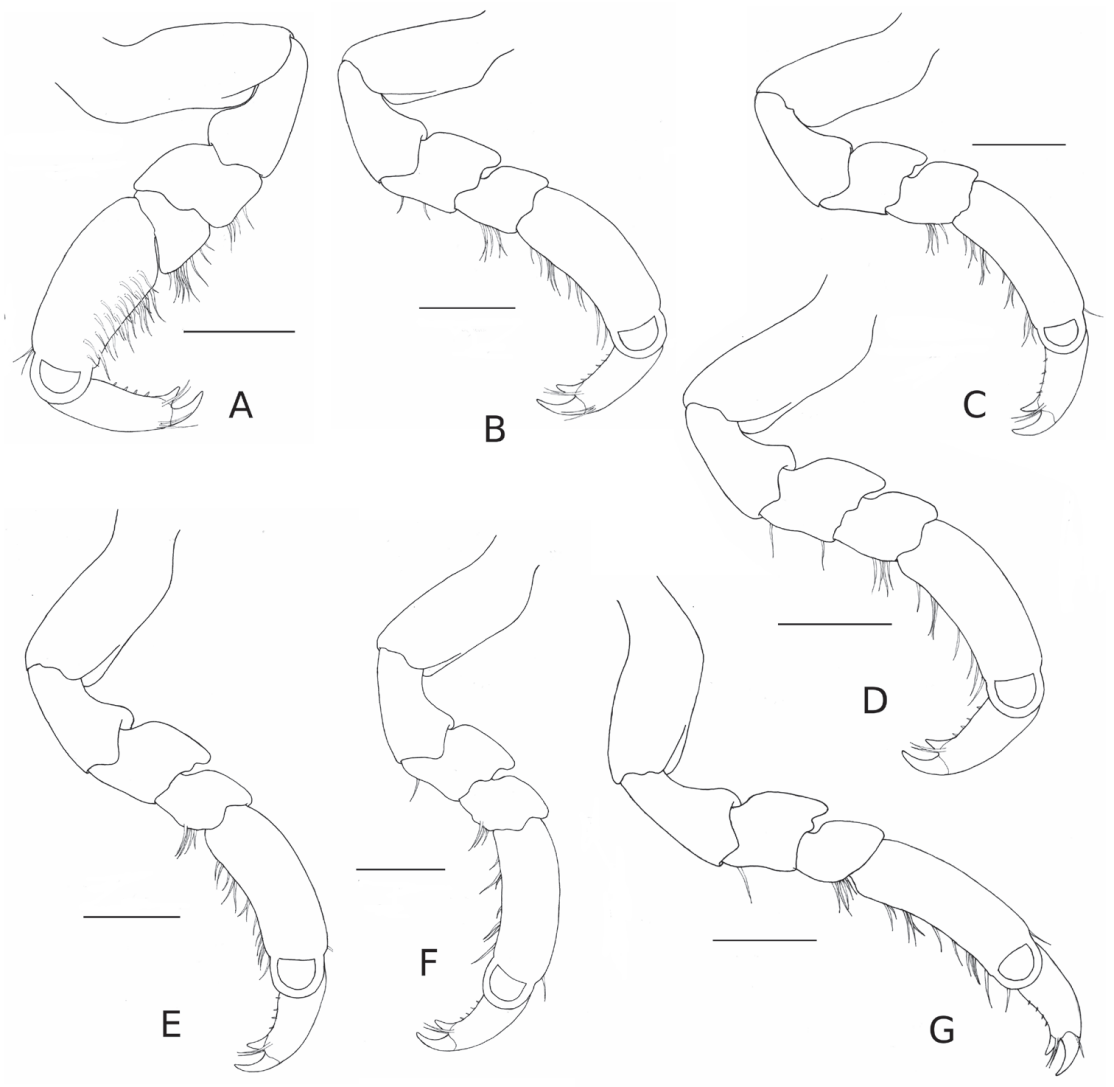
*Stenosoma stephenseni*, sp. n., holotype: **A** pereopod I **B** pereopod II **C** pereopod III **D** pereopod IV **E** pereopod V **F** pereopod VI **G** pereopod VII. Scale bars are 500 µm.

*Pereopods* I–VII ambulatory (Figure 2), robust, with merus 1.2 times as wide as long, and carpus 1.1 times as wide as long, terminating in a biungulate dactyl with simple setae; pereopod I with simple spines on inner surface of propodus, and weak setation on ventral margin; pereopods II–VII subsimilar; pereopods II and VI with 8–12 palmate setae on distal margin of propodus.

**Figure 3.**
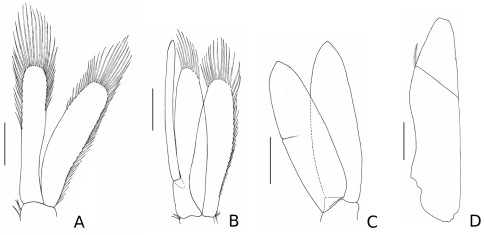
*Stenosoma stephenseni*, **sp. n.**, holotype: **A** pleopod I **B** pleopod II **C** pleopod III **D** uropod. Scale bars are 500 µm.

*Ventral penis smooth. Pleopods* I–II rami with plumose marginal setae (Figure 3); pleopod II with long appendix masculina, extending beyond endopod by more than one fifth of its length, but not beyond its apical spines, apex distal inner margin serrated, with five minute spines; pleopods III–V 1.1 times longer and 1.2 times wider than I–II, without setae. *Uropod:* uniramous, with small plumose seta on lateral distal angle of peduncle.

#### Etymology.

The epithet honours Knud Hensch Stephensen (1882–1947), former curator of the crustacean collections at the ZMUC, who first noticed that some specimens he placed in *Stenosoma acuminatum* were likely to be a new species from the Mediterranean (Stephensen, 1915).

#### Discussion.

The material from *Thor* campaigns in 1908–1810, originally described by Stephensen (1915) fits in well with the present description of *Stenosoma stephenseni* sp. n. (see figures from Stephensen, 1915: 15–16). In particular, the male appendix masculina (also drawn in Stephensen’s figures) leaves no doubt on the taxonomic status of both specimens.

There are three sympatric species with which *Stenosoma stephenseni* sp. n. can be confounded: *Stenosoma nadejda* (Rezig, 1989), *Stenosoma mediterraneum* (Rezig, 1989) and *Stenosoma capito* (Rathke, 1837). *Stenosoma stephenseni* sp. n. can be easily distinguished from all three species, as these have a mid-dorsal tubercle on the cephalon, one pair of lateral tubercles on the first two (*Stenosoma capito*) or three (*Stenosoma nadejda* and *Stenosoma mediterraneum*) pereonites, and more slender pereopods, with carpus and merus at least 1.5 times as long as wide. The appendix masculina does not extend beyond the apical margin of the endopod in *Stenosoma nadejda* (see Rezig 1989: 72), and extends beyond the apical margin of the endopod by 0.05 and 0.14 of its length in *Stenosoma mediterraneum* and *Stenosoma capito*, respectively. However, in the latter two species, the appendix masculina reaches the tip of the apical spines of the endopod (see Rezig 1989: 49, 65), whereas in *Stenosoma stephenseni* sp. n. it does not (Figure 3B).

As discussed below, the inclusion of Stephensen’s specimens labeled “*Stenosoma acuminatum”* in *Stenosoma stephenseni* sp. n**.** has implications for the distribution of *Stenosoma acuminatum*. According to (Naylor (1972, 1990), *Stenosoma acuminatum* ranges from the southwest coasts of Britain to the Mediterranean, Adriatic and Black Sea. However, no factual information (reference, site/date) is given for the presence of this species in the Mediterranean. Stephensen’s (1915) record remains as the only published and verifiable record of *Stenosoma acuminatum* in the Mediterranean.

After the description of *Stenosoma acuminatum* by Leach (1814), many authors opted to synonymise it with *Stenosoma appendiculatum* (Risso, 1826) or *Stenosoma capito* (Rathke, 1837). White (1847: 95), in his “List of the specimens of Crustacea in the collection of the British Museum”, listed a single specimen of *Idotea acuminata* from England (Leach’s own *Stenosoma acuminatum* from Devon, see also White 1850) and three specimens from Tripoli (unknown collector). As Leach never mentioned any material other than the one from Devon in his descriptions of *Stenosoma acuminatum* (Leach, 1814, 1815), the specimens from Tripoli must have been acquired later.

Bate and Westwood (1868: 394) re-described *Idotea acuminata* from the British Isles, basing their drawings and description on Leach’s specimen, but included “*Idotea capito*” from the Black Sea (attributed to Rathke 1937) in the list of synonyms. Thus, although they did not mention explicitly the Mediterranean, their popular reference clearly led the unaware reader to infer the presence of *Stenosoma acuminatum* in that region. Gourret (1891) corrected the error of Bate and Westwood (1868), but subsequent authors acknowledged their synonymy (e.g. Carus 1885; Stebbing 1893; Gerstaecker 1901; Monod 1923), always referring the presence of *Stenosoma acuminatum* in both the Mediterranean and the Atlantic. Yet, none of these works added a single new record of *Stenosoma acuminatum* from the Mediterranean, data being copied from earlier literature without further checking of taxonomic consistency. For example, Carus (1885) lists *Idotea acuminata* from the Mediterranean, synonymising it with “*Stenosoma acuminatum* Leach, *Idotea capito* Rathke, *Leptosoma lanceolatum* Risso, *Idotea lanciformis* Risso”, and ranging from “Mare Brittanicum” (data taken from Leach, 1814), “Pontus Euxinus” (Black Sea, data taken from Rathke 1837), Nice (data taken from Risso 1816), and Lissa, Lesina and Curzola (Croatia, Adriatic) which are records of *Idotea capito* (=*Stenosoma capito*) from Heller (1866).

Neither Miers (1881) nor Collinge (1917) helped in eliminating this confusion. Miers (1881) united all described *Stenosoma* species (except *Stenosoma carinatum*) under a single species: *Idotea acuminata*. However, he mentioned that “This is a very variable species, and I have been obliged to unite under one name several types that have usually been considered distinct”. He correctly placed the specimens from Tripoli belonging to the collections of the British Museum in the variety “*appendiculata”*, which he synonymised with *Stenosoma appendiculatum* (Risso, 1826). Collinge (1917) who did not examine any British specimens of *Stenosoma acuminatum*, copied literally the description of Miers (1881), along with its presumed distribution (Mediterranean, Adriatic, Black Sea and Atlantic, up to Scotland). These inaccuracies made their way into popular references (Naylor 1972, 1990), and although some authors questioned the presence of *Stenosoma acuminatum* in the Mediterranean (Amar 1957; Prunus and Pantoustier 1976; Junoy and Castelló 2003), the record of Stephensen (1915) has always been there to attest to the contrary.

By including the two specimens from the campaigns of the Thor (Stephensen 1915) in *Stenosoma stephenseni* sp. n. the only published and verifiable record of *Stenosoma acuminatum* in the Mediterranean is eliminated. Other published records (e.g. Graeffe 1902; Argano and Campanaro 2011) should be checked if collections are available. These are likely to be misidentifications of *Stenosoma appendiculatum*, as is the case of the unpublished record of A. Dohrn from Naples (1957-06-16), labeled “*Synisoma acuminata* Leach”, and deposited at the Stazione Zoologica Anton Dohrn. Specimens can be observed online (see movie for CRU072 at http://szn.i.hosei.ac.jp/HTML/Prep_list.php?Family=Idoteidae&ListType=icon). Their pereon margins are clearly serrated (triangular coxal plates) and the pleotelson shape is like an ink pen nib, two features characteristic of *Stenosoma appendiculatum*.

##### A note on *Idotea angustata* Lucas, 1849

During this work, the description of *Idotea angustata* Lucas, 1849 came to our attention. This species was described from Algiers (Algeria), and judging from its original description, clearly belongs to the genus *Stenosoma*, together with *Idotea carinata* Lucas, 1849*.* Carus (1885) included Lucas’ record in his list of the Mediterranean fauna, but since then *Idotea angustata* has never been used as a valid name again. Some authors synonimised it with *Stenosoma acuminatum* (e.g., Miers 1881), others with *Stenosoma capito* (Monod, 1925; Kussakin, 1982). Both the drawing and the description of *Idotea angustata* bear some similarities with *Stenosoma stephenseni* sp. n. but also with three other sympatric species: *Stenosoma mediterraneum* (Rezig, 1989), *Stenosoma nadejda* (Rezig, 1989), and *Stenosoma capito* (Rathke, 1837)

Lucas refers that “La tête est légèrement gibbeuse” [the head is slightly convex] and that “Les organes de la locomotion sont courtes et assez robustes” [the organs of locomotion are short and rather robust], but the lack of any reference to the presence/absence of lateral tubercles in the first pereonites, and the exact shape of the pleotelson and the protuberance of the cephalon make this description ambiguous. Hence the name *Idotea angustata* which, according to the rules of the ICZN, is available from Lucas (1849), could be either a junior subjective synonym of *Stenosoma capito* (Rathke, 1837) or a senior subjective synonym of *Stenosoma meditarraneum* (Rezig, 1989) , *Stenosoma nadejda* (Rezig, 1989) or *Stenosoma stephenseni* sp. n. According to Rezig (1989), Lucas’ specimens were deposited at the MNHNP, but they could not be found there and currently there is no indication as to their present whereabouts (Danièle Defaye, *pers. comm.*). Unless these material is found, *Stenosoma angustata* (Lucas, 1849) has to be treated as a *nomen dubium*.

##### Key to the species of the genus *Stenosoma*

**Table:** 

1	Antenna with multiarticulated flagellum	2
–	Antenna with single clavated flagellar article	12
2	Pleotelson without anterolateral sutures in either dorsal or lateral views	3
–	Pleotelson with one or three anterolateral sutures in dorsal or lateral views	8
3	Pleotelson with three suture lines in ventral view	4
–	Pleotelson without suture lines in ventral view	5
4	Cephalon with mid-dorsal tubercle; pereopods II–VII slender, with carpus and merus longer than wider; pereonites I–III with lateral tubercles	*Stenosoma nadejda* (Rezig, 1989)
–	Cephalon smooth, domed; pereopods II–VII robust, with carpus and merus slightly wider than longer; pereonites I–III smooth	S. stephenseni sp. n.
5	Cephalon with a mid-dorsal tubercle or spine	*Stenosoma wetzerae* (Ormsby, 1991)
–	Cephalon smooth	6
6	Pereon sides straight and parallel, coxal plates barely visible from above; pleotelson sides narrowing fairly evenly to an acute terminal projection	7
–	Pereon sides appearing serrated, coxal plates triangular in dorsal view; pleotelson shape like an ink pen nib	*Stenosoma lancifer* (Miers, 1881)
7	Antenna large, flagellum with more than seven articles	*Stenosoma acuminatum* Leach, 1814
–	Antenna short, flagellum with 5–7 articles	*Stenosoma pacificum* (Nunomura, 1974)
8	Pleotelson with one anterolateral suture in dorsal or lateral views	9
–	Pleotelson with three anterolateral sutures in dorsal or lateral views	10
9	Cephalon with a bilobed mid-dorsal tubercle; pereonites bearing a mid-dorsal spine	*S spinosum* (Amar, 1957)
–	Cephalon smooth; body with dorsal carina	*Stenosoma appendiculatum* (Risso, 1826)
10	Dorsal surface of anterior pereonites with tubercles; pleotelson narrow	11
–	Dorsal surface of anterior pereonites smooth, not tuberculate; pleotelson wide, shield-shaped (about 1.2 times as long as wide)	*Stenosoma carinatum* (Lucas, 1849)
11	Pereon with a mid-dorsal carina; one pair of lateral tubercles on the first two pereonites; pleotelson shape like an ink pen nib, with three anterolateral sutures visible laterally only	*Stenosoma capito* (Rathke, 1837)
–	Pereon without carina; one pair of lateral tubercles on the first three pereonites; pleotelson sides narrowing fairly evenly to an acute terminal projection, with three anterolateral sutures visible in dorsal view	*Stenosoma mediterraneum* (Rezig, 1989)
12	Pleotelson wide (length about 1.2 times width), shield-shaped, first suture larger than others	*Stenosoma bellonae* (Daguerre de Hureaux, 1968)
–	Pleotelson narrow (length equal to or more than 1.5 times width), lateral sutures short, all of the same length	13
13	Cephalon smooth; body without dorsal carina; maxilliped with one coupling hook	*Stenosoma raquelae* (Hedo & Junoy, 1999)
–	Cephalon with a bilobed mid-dorsal tubercle; body with dorsal carina; maxilliped with two coupling hooks	*Stenosoma albertoi* (Castellanos & Junoy, 2005)

## Supplementary Material

XML Treatment for Stenosoma

XML Treatment for Stenosoma stephenseni

## References

[B1] Amar R (1957) Sur un nouveau *Synisoma* méditerranéen (Isopoda Valvifera). Récueil des Travaux de la Station Marine d’Endoume 21: 74-79.

[B2] Argano R Campanaro A (2011) Isopoda. In: Relini G. (Ed) Checklist della fauna marina Italiana, http://www.sibm.it/CHECKLIST/principalechecklistfauna.htm [accessed 3.III.2011]

[B3] Bate CS Westwood JO (1868) A history of the British sessile-eyed Crustacea. Vol. II. John van Voorst, London, 536 pp.

[B4] Carus JV (1885) Prodromus faunae Mediterraneae. Vol I: Coelenterata, Echinodermata, Vermes, Arthropoda. E. Schweizerbart’sche Verlagshandlung, Stuttgart, 524 pp.

[B5] Castellanos C Junoy J (2005) *Synisoma albertoi*, a new species from the Strait of Gibraltar (southern Spain) with a key to known species of the genus (Crustacea: Isopoda: Idoteidae). Journal of the Marine Biological Association of the UK 85: 1461-1466. 10.1017/S0025315405012646

[B6] Collinge WE (1917) A revision of the British Idoteidae, a family of marine isopods. Transactions of the Royal Society of Edinburgh 51: 721-760.

[B7] Desmarest AG (1825) Considérations générales sur la classe des crustacés: et description des espèces de ces animaux, qui vivent dans la mer, sur les côtes, ou dans les eaux douces de la France. F.G. Levrault, Paris, 94 pp.

[B8] Dollfus A (1894) Les Idoteidae des côtes de France. Feuille des Jeunes Naturalistes 289: 1-5.

[B9] Dollfus A (1896) Les Idoteidae des côtes de France. Feuille des Jeunes Naturalistes 292: 53-56.

[B10] Gerstaecker A (1901) Crustacea. In: Ortmann AE (Ed) Die Klassen und Ordnungen der Arthropoden Vol. 5. C F Winter’sche Verlagshandlung, Leipzig, 1319 pp.

[B11] Gourret P (1891) Les Lemodipodes et les Isopodes du Golfe de Marseille. Annales du Musée d’Histoire Naturelle de Marseille-Zoologie 4: 1-38.

[B12] Graeffe E (1902) Uebersicht der Fauna des Golfes von Triest. V Crustacea. Arbeiten aus dem Zoologischen Instituten der Universität Wien und der Zoologischen Station in Triest 13: 33-80.

[B13] Heller C (1866) Carcinologische. Beiträge zur Fauna des Adriatischen Meeres Verhandlungen der Zoologisch-Botanischen Gesellschaft in Wien 16: 723-760.

[B14] ICZN (1999) International code of zoological nomenclature 4th ed. International Trust for Zoological Nomenclature, London, 306 pp.

[B15] Issel R (1912) Richerche di etologia sull’ isopodo tubiculo *Zenobiana prismatica* (Risso). Archives de Zoologiee Expérimentale et Générale 51: 479-500.

[B16] Junoy J Castelló J (2003) Catálogo de las especies ibéricas y baleares de isópodos marinos (Crustacea: Isopoda). Boletin del Instituto Español de Oceanografia 19: 293-325.

[B17] Kussakin OG (1982) Marine and brackish-water Isopoda of the cold and temperate waters of the Northern Hemisphere. Vol II. Suborders Anthuridea, Microcerberidea, Valvifera and Tyloidea. Opredeliteli po Faune SSSR 131: 1-464.

[B18] Latreille PA (1829) Les crustacés, les arachnides et les insects. In: Cuvier G. (Ed) Le Règne Animal, distribué d’après son organisation, pour servir de base à l’histoire naturelle des animaux et d’introduction à l’anatomie comparée. Vol. 4 Deterville, Paris, 1–584.

[B19] Leach WE (1814) Crustaceology. In: Brewster D (Ed). The Edinburgh Encyclopaedia. Vol. 7. Blackwood, Edinburgh: 383-437.

[B20] Leach WE (1815) A tabular view of the external characters of four classes of animals, which Linné arranged under Insecta; with the description of the genera comprising three of these classes into order, etc., and descriptions of several new genera and species. Transactions of the Linnean Society of London 2: 306-400.

[B21] Lucas AH (1849) Histoire naturelle des Animaux Articulés. Exploration scientifique de l’Algérie pendant les années 1840, 1841, 1842. Sciences Physiques Zoologie 1: 1-403.

[B22] Miers EJ (1881) Revision of the Idoteidae, a family of sessile-eyed Crustacea. Journal of the Linnean Society of London 16: 1-88. 10.1111/j.1096-3642.1881.tb02274a.x

[B23] Milne-Edwards M (1840) Histoire naturelle des crustacés, comprenant l’anatomie, la physiologie et la classification de ces animaux. Vol. 3. Libraire Encyclopédique de Roret, Paris, 638 pp.

[B24] Monod T (1923) Prodome d’une faune des Tanaidacea et des Isopoda (Excl. Epicaridea) des côtes de France (Excl.Méditerranée). Société des Sciences Naturelles de Charente Inférieure 37: 19-125.

[B25] Monod T (1925) Tanaidacés et Isopodes aquatiques de l’Afrique occidentale et septentrionale. 1ère partie: Tanaidacea, Anthuridea, Valvifera. Bulletin de la Société des Sciences Naturelles et Physiques du Maroc 5: 61-85.

[B26] Naylor E (1972) British marine isopods: keys and notes for the identification of the species. Synopsis of the British Fauna Vol. 3. Academic Press, London, 86 pp.

[B27] Naylor E (1990). Isopoda. In: Hayward PJ & Ryland JS (Eds). The Marine Fauna of the British Isles and North-west Europe, Vol. 1. Oxford, Clarendon Press: 387-405.

[B28] Nobre A (1903) Subsídios para o estudo da fauna marinha do norte de Portugal. Annaes de Ciencias Naturaes 8: 37-94.

[B29] Norman AM (1904) British Isopoda of the families Aegidae, Cirolanidae, Idoteidae, and Arcturidae. Annals and Magazine of Natural History 7: 430-450. 10.1080/03745480409443033

[B30] Norman AM Scott T (1906) The Crustacea of Devon and Cornwall. William Wesley and Son, London, 232 pp.

[B31] Prunus G Pantoustier G (1976) Le genre *Synisoma* Collinge (Isopoda Valvifera) en Tunisie: Description de *Synisoma teissieri* nov. sp. Crustaceana 31: 259-266. 10.1163/156854076X00044

[B32] Rathke H (1837) Zur Fauna der Krym. Mémoires de l’Academie Impériale des Sciences de St Pétersbourg 3: 291-454.

[B33] Rezig M (1989) Les idoteidae du genre *Synisoma* Collinge (Isopoda, Valvifera) du littoral tunisien. Revue de la Faculté des Science de Tunis 4: 29-80.

[B34] Risso A (1816) Histoire naturelle des Crustacés des environs de Nice. Librairie Grecque-Latine-Allemand, Paris, 176 pp.

[B35] Risso A (1826) Histoire naturelle des principales productions de l’Europe Méridionale. Vol. 5. F. G. Levrault, Paris, 403 pp.

[B36] Stebbing TRR (1893) A history of Crustacea. Kegan Paul, Trench, Trubner & Co, London, 466 pp.

[B37] Stephensen K (1915) Isopoda, Tanaidacea, Cumacea, Amphipoda (Excl. Hyperiidea). Report Danish Oceanographical Expeds 1908–1910 to Mediterranean and adjacent seas 2 (Biology): 1–53.

[B38] Tattersall WM (1911) Die Nordischen Isopoden. In: Brandt K Apstein C (Eds). Nordisches Plankton Vol. 3 Crustacea. Verlag von Lipsius and Tischer, Kiel: 181-314.

[B39] Xavier R Zenboudji S Lima FP Harris DJ Santos AM Branco M (2011) Phylogeography of the marine isopod *Stenosoma nadejda* (Rezig, 1989) in North African Atlantic and western Mediterranean coasts reveals complex differentiation patterns and a new species. Biological Journal of the Linnean Society 104: 419-431. doi: 10.1111/j.1095-8312.2011.01718.x

[B40] White A (1847) List of the specimens of Crustacea in the collections of the British Museum British Museum, London, 157 pp.

[B41] White A (1850) List of the specimens of British Animals in the collection of the British Museum (Part IV) Crustacea. British Museum, London, 141 pp.

